# Shaping futures through gendered lenses: secondary school students’ perceptions on gender roles and career choices

**DOI:** 10.3389/fpsyg.2026.1658581

**Published:** 2026-02-09

**Authors:** Zeynep Demirtaş, Aynur Rabia Özbek, Ömer Faruk Vural

**Affiliations:** 1 Sakarya University, Faculty of Education, Department of Education Sciences, Sakarya, Türkiye; 2 Sakarya University, Institute of Educational Sciences, Department of Education Sciences, Sakarya, Türkiye

**Keywords:** gender roles, career choices, occupational preference, traditional gender perceptions, social norms and employment

## Abstract

**Introduction:**

The qualitative research study aims to determine secondary school students’ perceptions of how gender roles affect their career choices.

**Methods:**

In this study, the qualitative research design “phenomenology” was used. The study group was determined according to the maximum diversity sampling technique, one of the purposeful sampling methods. The study group consisted of 100 students attending different types of secondary schools in the province of Sakarya. A semi-structured interview form prepared by the researchers was used as the data collection tool in the study. This form consisted of four sections: demographic information, educational information, questions related to career preferences, and gender roles. Content analysis was used to analyze the data, and student opinions were included.

**Results:**

The study’s findings revealed that the students’ thoughts and perceptions were consistent with traditional gender roles and that this effect was evident in their career choices. Male students want to work in a profession that offers high pay and career opportunities. In contrast, female students want to work in a profession that does not interfere with their domestic responsibilities, especially motherhood. The students who participated in the study believe they will be more successful and more easily employed in occupational groups associated with traditional gender roles in the workplace.

**Discussion:**

The students explained their career choices within the same frameworks and boundaries, using traditional gender perceptions.

## 1 Introduction

Career choice is a critical process that can have long-term impacts on individuals’ lives. In this process, gender roles may play a decisive role in shaping individuals’ interests, abilities, and professional goals (Aslan Çetin, 2021; Dahiya, 2024). Social structures create specific roles and expectations for women and men, which can limit or guide individuals’ career orientations (Ecevit, 2003). Particularly during adolescence, individuals’ perceptions regarding career choices are largely shaped by these norms.

In Turkey, where traditional family and social structures remain highly influential, career decisions are often intertwined with gender expectations and educational opportunities. According to the Turkish Statistical Institute (TÜİK, 2024), women’s labor force participation rate is 36.8%, compared to 72.0% for men, reflecting the strong influence of gender norms on employment and education. The Global Gender Gap Report (WEF World Economic Report, 2024) ranks Turkey 127th out of 146 countries overall and 133rd in economic participation, underscoring the persistence of gender inequality. Historically, Turkish women’s access to higher education and professional life has been shaped by urban–rural disparities, patriarchal values, and unequal caregiving expectations (Köseoğlu, 2017; Özveren and Dama, 2022). These contextual factors make the study of gender and career choice in Turkey particularly salient for understanding how culturally embedded gender roles affect adolescents’ career perceptions.

Moreover, the global COVID-19 pandemic has intensified existing gender inequalities and reinforced traditional patriarchal norms in work and family life. Women disproportionately shouldered additional caregiving, household, and remote schooling responsibilities during the pandemic, while facing disruptions in their professional trajectories (Dinella et al., 2023a; Dinella et al., 2023b; Fulcher et al., 2023). This retrenchment of gender roles highlights how societal expectations of women’s work and family responsibilities can limit opportunities and shape career perceptions, making it particularly relevant to investigate how these norms influence adolescents’ occupational choices. Situating the present study within this global context underscores that understanding gendered career preferences is not only a reflection of local traditions but also a response to worldwide phenomena that have exacerbated disparities in work, family, and social roles.

Gender stereotypes lead to the division of occupations into “male” and “female” professions. Women are often directed toward fields such as caregiving and education, while men are concentrated in technical or managerial positions, reflecting these stereotypes (Epstein, 2022; Karamanlı, 2019; Lozano et al., 2022). While these studies were largely conducted in Western contexts (e.g., United States and Europe), similar patterns have been identified in Turkey, where teaching, nursing, and child development are perceived as feminine occupations, and engineering and management are perceived as masculine (Ecevit, 2003; Tarhan et al., 2014; Bulut and Başfırıncı, 2021). The present study aims to examine how students’ perceptions of gender roles are related to their occupational preferences in the Turkish secondary education context.

### 1.1 Sex, gender, and gender roles

Sex refers to the classification of individuals as female or male based on their innate physiological and genetic characteristics (e.g., chromosomes, reproductive anatomy) (Deaux, 1985; Dökmen, 2021; Lips, 2001). In contrast, gender pertains to the social roles, behavioral patterns, and expectations attributed to individuals based on their sex; this construct is shaped in accordance with cultural norms (Dökmen, 2021; Purvis, 1987). Therefore, gender is not a fixed or biological reality; it is a conceptual framework that is learned, internalized, and subject to change over time (Greenglass, 1982; Lindsey, 2015).

Gender roles encompass societal rules that dictate which behaviors are considered “appropriate” for individuals. These roles are learned through messages received in the family, educational institutions, media, and broader social environments (Powell and Greenhaus, 2010). For instance, girls are generally taught roles such as caregiving and being graceful, whereas boys are encouraged to be strong, leaders, and to suppress their emotions (Akgül, 2022; Saraç, 2013).

In Turkey, these gendered expectations are transmitted early in childhood through family socialization and the education system, where textbooks and media representations continue to emphasize traditional gender divisions (Okan, 2024; Ünal et al., 2017). For example, female characters are often depicted in domestic or nurturing roles, whereas male figures appear in public and decision-making contexts (Aşkun and Erkoyuncu, 2023). Such cultural representations contribute to the internalization of gender norms and influence how young people perceive “appropriate” career paths.

Society constructs the social dimension of gender identity by assigning different roles to women and men (Dökmen, 2021). These roles significantly influence how individuals define themselves and which occupations, behaviors, or lifestyles they adopt. This social structure guides the opportunities, responsibilities, and choices individuals encounter throughout their lives, making gender a determining factor in critical decisions, particularly in career choices. Over time, these roles are internalized and become part of individuals’ self-concept, influencing their perceived interests, competencies, and career aspirations (Eccles, 2011; Diekman et al., 2010). Thus, gender functions as both a social category and a psychological schema guiding occupational decision-making.

### 1.2 The relationship between career choice and gender roles

Career choice is a critical process that affects an individual’s lifestyle, social status, and psychological well-being, and should align with their interests and abilities (Atlı, 2012; Kuzgun, 2014; Niles and Harris Bowlsbey, 2013; Yeşilyaprak, 2000). This process is shaped not only by individual preferences but also by societal norms and gender roles (Baltaş, 1993; Deniz, 2001). Gender roles guide individuals’ career choices into specific patterns. Men are generally directed toward high-status and technical occupations in the public sphere, whereas women are steered toward professions considered compatible with domestic and family life (Bulut and Başfırıncı, 2021; Hartmann, 2008). Even when women participate in the workforce, they continue to bear household and caregiving responsibilities (Çuhadar, 2024). This pattern is especially evident in Turkey, where women spend over 4 hours per day on unpaid domestic and caregiving work, compared to men, and their labor force participation remains considerably lower than men’s (OECD, 2025). Such unequal divisions of labor shape not only adult employment but also adolescents’ perceptions of gender-appropriate careers.

Society expects individuals to make life and career choices that conform to gender roles (Charles and Bradley, 2009; Gökcan, 2018; Tarhan et al., 2014). Gender roles are a central concept in understanding individuals’ behaviors and career choices. Social Role Theory emphasizes that the behaviors of women and men are shaped by socially determined expectations and socialization processes (Eagly and Wood, 2012); this may lead to gender-based differences in career preferences, which can vary across cultural contexts. According to this theory, the association of women with caregiving and domestic responsibilities, and men with economic and leadership roles, plays a decisive role in shaping individuals’ occupational choices and social perceptions. This division of labor corresponds closely to what has been termed the breadwinner–caregiver model (Deutsch, 2007; Eagly and Wood, 2012). The persistence of this model across cultures has reinforced occupational gender segregation, as caregiving professions (e.g., teaching, nursing) are coded as feminine and leadership or technical fields are coded as masculine (Ridgeway and Correll, 2004). In societies like Turkey, where traditional family structures remain influential, this model continues to shape young people’s perceptions of which professions are “appropriate” for men and women, thereby limiting individual autonomy in career decision-making. The intersectionality approach, on the other hand, addresses inequalities arising from the intersection of multiple identity categories (e.g., gender, class, socioeconomic status) (Crenshaw, 1989). This perspective enables the evaluation of gender effects in relation to economic, cultural, and social contexts. In the Turkish context, this theoretical framework provides an important foundation for examining the influence of students’ perceptions of gender roles on their career choices and for revealing the unique reflections of gender inequality in education, employment, and social life. This situation creates an obstacle to the full utilization of individual potential. Our study uses this distinction consistently and focuses on perceived gender roles and their relation to career preferences.

### 1.3 Gender-based division of labor and discrimination

The persistence of traditional gender ideologies sustains structural divisions in both family and labor contexts, which are best understood through the concept of a gendered division of labor. Sex categorizes individuals by their biological characteristics, while gender captures the socio-cultural meanings attached to being female or male. Societies often map these meanings onto work, constructing a gendered division of labor in which certain occupations are perceived as “women’s” or “men’s” jobs. Gender also refers to the roles that individuals assume in society as women or men, shaped by cultural norms and values (Bhasin, 2003; Dökmen, 2021; Oakley, 1985). These roles guide career preferences and reinforce a gender-based division of labor. Such prevalent associations strengthen the idea that some professions are more suitable for women and others for men (Sağıroğlu, 2021).

Gender roles are internalized by individuals through socialization agents such as education and media, making them a determining factor in career choices (Ecevit, 2003; Ünal et al., 2017). Although women’s participation in the workforce has increased, the construction of occupations based on masculine values results in women encountering discrimination in professional life (Yerlikaya, 2019). In the workplace, this discrimination often manifests as horizontal and vertical occupational segregation. Horizontal segregation refers to the concentration of women in specific professions and their exclusion from other fields (Urhan and Etiler, 2011). An example of this is the predominance of women in traditionally “female-appropriate” occupations such as nursing and teaching (Parlaktuna, 2010). Vertical segregation, on the other hand, occurs when women occupy lower-status positions within the same occupational group; the disadvantages women face in career advancement due to marriage and motherhood are key factors in this phenomenon (Aksu et al., 2013; Ecevit, 2011; ILO, International Labour Organization, 2008). Gender-based discrimination affects not only occupational choice but also wage equality. Women are paid less than men for performing the same work and are valued less despite possessing equivalent qualifications (Bulut and Kızıldağ, 2017; Erikli, 2020).

In patriarchal societies like Turkey, gender norms result in women being disadvantaged across many areas, from education to professional life (Köseoğlu, 2017; Köyüstü Erdeniz, 2017). This directly influences decisions regarding the types of work women can perform and the fields in which they can pursue career goals. Household chores and childcare are socially perceived as “natural” duties for women, whereas men are encouraged to participate in economic activities (Özveren and Dama, 2022; TÜİK, 2022). Therefore, gender inequality in Turkey persists as a multidimensional issue that limits women’s opportunities in professional and social life through its economic, cultural, and structural dimensions (Aşkun and Erkoyuncu, 2023; Küçük Aksu, 2022). Gender identity is shaped from childhood through family and educational institutions, where different roles are assigned to girls and boys (Okan, 2024). These roles are also reflected in career choices, constraining individual decision-making. According to the World Economic Forum’s 2024 Global Gender Gap Report, Turkey ranks 127th out of 146 countries in gender equality, and 133rd in terms of economic opportunities. The report predicts that achieving full gender equality will require 134 years (WEF World Economic Report, 2024).

Studies (Açar and Dilbilir, 2024; Artar and Fildiş, 2021; Dalagan, 2024; Koşar Taş, 2021; Okan, 2024; Özsoy, 2024; Özveren and Dama, 2022) emphasize that gender inequality exists in Turkish society and that women are negatively affected. Research on gender and career choice in Turkey has mostly focused on adults, university students, or specific occupational groups (Altıparmak and Kök, 2025; Asa, 2023; Demir, 2020; Filiz and Güzelyurt, 2017; Gül and Öcal, 2021; Köseoğlu, 2017; Soylu and Esen, 2022).

This study evaluated the impact of gender on the career preferences of 11th and 12th-grade secondary school students. The research examined the question, “How do gender roles affect career choices according to the views of secondary school students?” Students’ thoughts and decisions regarding career choices were analyzed in the context of gender. This study presents original findings on secondary school students’ perceptions of traditional gender roles in the Turkish educational context, revealing their reflections on career choices. By examining how gender perceptions influence career choices, the study aims to raise awareness and contribute to strategies that support students’ freedom of choice and a more gender-equal society.

## 2 Method

### 2.1 Research method

This qualitative research study examines secondary school students’ perceptions of whether gender roles affect their career choices and, if so, how. Qualitative research is a method applied to question, interpret, and understand the problem situation within its natural conditions (Yıldırım and Şimşek, 2018). This study used the “phenomenology” design among qualitative research designs. Phenomenology is a suitable method for examining phenomena that require in-depth and detailed information about a situation. The phenomenology design is used when there is awareness of a phenomenon, but more detailed and in-depth information is desired (Cypress, 2018; Yıldırım and Şimşek, 2018). The phenomenological design is an appropriate research method for examining complex issues such as the impact of gender roles on career choice, as it allows for in-depth and detailed examination of participants’ experiences and perceptions.

### 2.2 Research study group

The study group for this research consists of students attending different types of secondary education institutions in the province and districts of Sakarya, Turkey. Students were selected using the maximum diversity sampling method, one of the purposeful sampling methods, to achieve the research’s aim. Maximum diversity sampling is based on reflecting the diversity of individuals who may be involved in the problem to the maximum extent (Mertens, 2023; Yıldırım and Şimşek, 2018). In this context, diversity includes secondary education types, family structures with different income groups, and the regions where students live (province, district center, village, etc.), and the study group was formed by considering these elements. The study group comprises 100 students in the 11th and 12th grades of different secondary education schools.

In Turkey, students are placed in different high school types based on their High School Placement Exam (LGS) scores and personal preferences, which can influence the gender composition of certain departments. Vocational and technical high schools have separate departments, and within these departments, female and male students are often separated due to societal norms and educational track requirements.

### 2.3 Demographic information of participants

The research was conducted with the participation of 11th and 12th-grade secondary education students, who are in the learning period when they begin to decide on their career choices. In Turkey, career decisions at this stage are influenced by a combination of interest-based preferences and the national examination system, particularly the High School Placement Exam (LGS) and the Higher Education Institutions Exam (YKS). Students typically choose fields of study in high school that align with their career aspirations, but these choices are often constrained by exam scores and school quotas. Seventy percent of the students interviewed were 17 years old, and 30% were 16 years old. The reason for interviewing mainly 17-year-old students is that by the final year of high school in Turkey, students have largely determined their career paths based on their academic track and university preparation, and they are transitioning to higher education. While the number of female and male students in the 11th grade participated in the study was equal, 48.5% of the 12th grade students were male (34 people) and 51.5% were female (36 people). A total of 100 students participated in the study, 51 of whom were female and 49 of whom were male. Table 1 shows the distribution of participants according to secondary school type and gender.

**Table 1:** Percentage of participants by secondary school type and gender.

Secondary school type	Gender	Number	Sum	Percent
Anatolian high school	Female	14	27	27%
	Male	13		
Vocational and technical Anatolian high school	Female	17	35	35%
	Male	18		
Science high school	Female	6	13	13%
	Male	7		
Imam Hatip high school	Female	14	25	25%
	Male	11		
Sum		100	100	100

According to the school types, 27 students (27%) from Anatolian high schools, 35 students (35%) from vocational and technical Anatolian high schools, 13 students (13%) from science high schools, and 25 students (25%) from Imam Hatip high schools participated in the study. Most of the students interviewed (35%) were from vocational and technical Anatolian high schools. The lower participation rate of 13% from science high schools is because the interview process reached data saturation, as determined by the research team when no new themes emerged from consecutive interviews. Data saturation was achieved across all school types, indicating that sufficient and comprehensive data were collected from students in every category of secondary education. In this study, the terms “female” and “male” are used to denote biological sex, as obtained from demographic questions. When discussing social roles, the terms “woman” and “man” are used.

### 2.4 Data collection tools

In phenomenological research, it is important to reach individuals who experience and reflect on the phenomenon that is the focus of the research. The primary data collection method is interviews (Mertens, 2023). Interviews provide researchers with the opportunity to explore experiences and meanings related to phenomena through interaction, flexibility, and probing (Yıldırım and Şimşek, 2018). In this study, a detailed and extensive literature review was conducted on gender, the concept of profession, and career choice during the design phase of the interview form. Basic questions appropriate to the purpose of the research were determined. More specific sub-questions supported the basic questions to encourage participants to explain their experiences, opinions, and thoughts in depth. Expert opinion was sought from two academics specialized in educational sciences and gender studies (faculty members at a public university in Turkey) regarding the appropriateness of the questions in relation to the purpose and problem of the research, and the questions were revised based on their feedback. A pilot application was conducted, the interview form was in draft form, and the final version was finalized. The interview form consists of four sections: the first section covers demographic information (gender, age, place of residence); the second section includes educational information (grade level, type of high school attended, field of study); the third section addresses career preferences; and the fourth section comprises open-ended questions related to career choice and gender. The questions regarding career preferences are as follows: To what extent did you consider the profession(s) you plan to pursue in the future when making your field choice? What are the main reasons for choosing these professions? Could you describe in your own words the factors that influenced your career choice? What characteristics would you like the profession you wish to work in to have? The questions related to career choice and gender are as follows: Do you think that certain professions are considered more suitable for women and others for men in society? How do you believe these perceptions affect career choices? Have such perceptions influenced your own choices? Do you think the societal expectations and responsibilities assigned to women and men affect career choices? How do you think family roles influence work preferences and success in professional life? What challenges do you think women and men face in the workplace? Do these challenges affect your career choices?

### 2.5 Data collection process

Data related to the research were collected through interviews with 11th and 12th-grade students attending different types of secondary schools in the districts of Hendek, Adapazarı, Serdivan, and Akyazı in the province of Sakarya, Turkey, between February 7 and May 15, 2022. All interviews were audio-recorded, and recordings were transcribed verbatim. Transcription was performed manually by the researchers; no automated transcription software was used. Approximately 15% of students declined to be audio-recorded, and for these cases, the interviewers transcribed responses manually during or immediately after the interview. The interviews lasted approximately 30–35 min.

### 2.6 Researcher reflexivity

In this study, the researchers played an active role in the data collection and analysis processes in accordance with the nature of the phenomenological design. Researcher 1 is a female, Turkish, PhD candidate in Educational Sciences, with professional experience in secondary education and a focus on gender studies. Researcher 2 is a male, Turkish, faculty member in Educational Sciences, with expertise in qualitative research and sociology of education. Both researchers’ professional backgrounds and awareness of gender equality may have shaped the interpretation of the data. The aim of the researchers was to gain an in-depth understanding of students’ perceptions regarding gender roles and career choices. During the interviews, the researchers endeavored to establish empathetic communication with the students, use non-directive open-ended questions, and minimize the influence of their own value judgments on the data. In this way, the impact of researchers’ biases or assumptions on the findings was limited as much as possible.

### 2.7 Data analysis

This study used content analysis to analyze the data obtained from the participants. Content analysis involves grouping similar data under specific concepts and themes and organizing and interpreting these groups in a way that is understandable to the reader (Yıldırım and Şimşek, 2018, p. 242). The data collected through interviews were transcribed and prepared for analysis. In the analysis process, themes were not predetermined; instead, codes were developed through careful examination of the data and then grouped based on their similarities to derive the themes. Thus, the analysis was conducted using a data-driven (inductive) content analysis approach based on the participants’ statements (Yıldırım and Şimşek, 2018). Each piece of data was carefully examined, and the most appropriate codes were assigned to the content. The coded data were reviewed, and the codes were classified into the same themes. These themes reflect the key issues and findings related to the impact of gender roles on career choice. Direct quotations supported the findings obtained. Participants were coded based on the order of the interviews using the letter “I” (short for Interviewee), followed by a number (e.g., I1, I2). The participants’ genders were indicated with “M” (male) and “F” (female), and the type of high school they attended was also included in the coding. For example, the code (I24, M, Vocational and Technical High School) indicates that interviewee number 24 is a male student attending a Vocational and Technical Anatolian High School.

In qualitative research, credibility, transferability, consistency, and confirmability are prioritized over validity and reliability (Mills, 2003). Regarding reliability, the researcher must participate in the data collection and take notes (Kozak, 2014). Additionally, providing detailed information about the research and data analysis process enhances the study’s credibility (Elo et al., 2014). In this study, the research process was explained in detail, and each stage of the study was specified, with care taken to ensure objectivity at every stage. During the coding process, the researchers coded independently. Then, the percentage of agreement between the codes was calculated. The agreement formula proposed by Miles and Huberman (1994) [Agreement = (Number of Common Codes/(Number of Common Codes + Number of Different Codes)) × 100] was used. As a result of the calculations, the coding agreement rate between the researchers was 89%. This rate is considered reliable. In cases of coding differences, the researchers reached a consensus. The findings were presented in tables and explained with numerical data (f). Additionally, the findings were supported by direct quotations from the participants’ views.

## 3 Findings

The views of female and male secondary school students on career choice are presented in Table 2.

**Table 2:** Opinions of female and male students regarding career choice.

Theme	Codes	Female (f)	Male (f)	Total (f)
Factors affecting career choice	Job opportunities	12	10	22
	Interest and ability	9	12	21
	Social Prestige	6	11	17
	University exams	10	2	12
	Career development	4	8	12
	Entrepreneurship	2	5	7
	Family guidance	7	1	8
	High earnings	5	9	14
Preferred working conditions	High paying	9	18	27
	Have my own business	8	13	21
	Promotion	3	8	11
	Let it be a workplace where my gender is the majority	15	0	15
	Let it be a job where I can work at a desk	14	3	17
	Let it be a job that I can manage/be actively in the field	2	7	9

In Table 2, female and male students’ views on career choice are coded under “Factors influencing career choice” and “Preferred working conditions.” When examining the factors influencing students’ career choices, it was observed that the order of importance differed between female and male students. In general, the opportunity to find a job is the most important factor for students (*f* = 22), followed by interest and ability (*f* = 21) and social prestige (*f* = 17). The frequency intensity of the interest, ability, and social prestige factors originates from male students in the table. From this perspective, it is necessary to examine the factors influencing the career choices of female and male students separately.

Male students’ most important factor in career choice is interest and ability. This is followed by social prestige, job opportunities, high-income, career development, the opportunity to start a business, university entrance exams, and family guidance. As can be seen, family guidance ranks last for male students, while social prestige ranks second, and high income ranks third. For men, the profession’s status in society and high income influence career choice. For female students, the most important factors influencing career choice are job opportunities, university entrance exams, interest and ability, family guidance, social prestige, high income, career development opportunities, and the opportunity to start a business. Family guidance ranks fourth among female students, while the opportunity to start one’s own business ranks last. Some of the students’ views are presented below.

“Men evaluate professions based on their interests and abilities. By continuously working and developing these interests and abilities, one can build a career” (G24, M, Vocational and Technical High School).

“If my income is high, if I have a job with social prestige, and if I have the opportunity to find a job, I do not care much about the other factors. Under these conditions, you love your job” (G29, M, Anatolian High School).

“In professional life, professions and titles are important. Not everyone with a title deserves respect, but society looks at the title and shows respect” (G35, F, Imam Hatip High School).

When examining the working conditions preferred by students in Table 2, most students (*f* = 27) stated that they wanted to work in a high-paying job. When it comes to work conditions based on gender, the most common view among women (*f* = 15) is “I want to work in a place where most of my coworkers are women,” and the second most common view (*f* = 14) is “I want a job where I can work at a desk.” For male students, the top three options are, in order, “high-paying,” “my own business,” and “offering promotion opportunities.” Regarding these views, the statements of Interviewer 31 and Interviewer 78 are as follows:

“If I have my own business, I will earn more. This is also very important in terms of social prestige. Such a job is very intense. From the perspective of family life, being in such an intense job does not have a positive social impact, but my opinion is that family life should not be prioritized” (G31, M, Vocational and Technical Anatolian High School).

“I want to work at a desk job so that I do not get too tired, especially regarding household responsibilities, so that I can be refreshed when I go home” (G78, F, Imam Hatip High School).

Some female students (*f* = 8) said they would like to have their own business. The reason behind this view, which contrasts with that of male students, is that it would allow them to devote more time to household chores. Interviewee 63 expressed this situation as follows:

Having my own business is my dream. If I am going to work, it will be in the form of setting up my office and doing my work, because this situation makes my life financially and spiritually easier. I can fulfill my household responsibilities, my work and home life will be organized, and I will be happy in the future” (G63, F, Science High School).

When examining these findings, some differences according to school type can be observed. Male students in vocational and technical high schools prioritize social prestige, high income, and entrepreneurship, whereas male students in Anatolian and science high schools consider their interests and abilities to be more decisive. Female students in science high schools prefer to establish their own businesses, valuing the balance between work and home life, while female students in Imam Hatip and Anatolian high schools tend to prefer desk-based jobs or workplaces with a majority of female colleagues.

When Table 3 is examined, students’ opinions on “The effect of social stereotypes on occupational choice” were coded as “the guiding effect of gender stereotypes on occupational preferences” (*f* = 43), “the effects of individuals’ gender roles on occupational choice” (*f* = 37), “society’s classification of professions as women’s professions and men’s professions” (*f* = 30), and “women and men feeling the pressure of society in their occupational choices” (*f* = 24). Students who evaluate their occupational preferences based on gender stereotypes believe that societal views on gender influence career choices, associating certain professions more with women and others more with men, which leads to a visible division of roles in the professional world. In this context, they expressed their views that gender stereotypes in society influence career choices in ways that are not always explicit but can be observed in certain professions, resulting in women being overrepresented in some fields and underrepresented in others. According to the students’ opinions in Table 3, it can be said that gender stereotypes constructed by society affect the choice of profession.

**Table 3:** The effect of social stereotypes on career choice.

Category	Codes	Female (f)	Male (f)	Total (f)
The effect of social stereotypes on occupational choice	The guiding effect of gender-stereotyped traits on career choices	23	20	43
	The influence of gender roles on career selection	17	20	37
	Society’s classification of professions as male or female	18	12	30
	Social pressure on men and women in occupational choices	15	9	24

When the students’ opinions on “the guiding effect of gender stereotypes on occupational preferences” are examined, it is seen that the students think that jobs such as desk work, professions that require a smiling face, and caring for patients and children are more suitable for women. The interviewees express this situation as follows.

“A sympathetic person with a smiling face can take any kind of job as a babysitter, for example, with her energy, and women are suitable for this” (I77, M, İmam Hatip High School).

“In a female nurse, the patient can feel safer and think they will be hurt less because women are friendlier. Since women have a maternal structure, professions where they feel maternal are more suitable for women. Men, on the other hand, tend towards professions with better incomes due to livelihood concerns” (I26, F, Anatolian High School).

When the “Effects of individuals’ gender roles on occupational choice” is analyzed, most students who participated in the research think that men should be oriented towards professions where they can earn high incomes, since men have the role of providing for the household. Even if women work, this is seen only as contributing to the home economy. In this context, women should work in jobs considered more appropriate for their gender roles and where they will not interrupt their responsibilities. Interviewee 72 explained this situation as follows.

“Women can choose a job like teaching, working hours are less, and they have vacations. The day and night of a woman working in an ambulance interfere with her day and night. For example, when this woman gets married and has a child, she gets into more trouble. When choosing a profession, family life should be at the forefront for women. Conversely, men want to work somewhere with a higher salary because they are responsible for providing for the household” (I72, F, Science High School).

When the opinions on “society’s classification of professions as women’s professions and men’s professions” were examined, it was seen that women’s responsibilities are to maintain order and cleanliness in the house and to take care of children, while men’s primary responsibilities are to provide for the house, that is, to work in a paid job and to do repair and renovation work. Accordingly, women should prefer jobs that do not interfere with motherhood and home responsibilities.

“She should plan her professional life. She can choose a job that allows her to care for the child, for example, she can work in a home office” (I17, M, Anatolian High School).

“For example, imagine a girl who is made to clean and cook at home. That girl can study cookery or astronomy; she is made to believe she has a talent for this and is directed to this field. Professions can be chosen according to the responsibilities at home. On the other hand, they both need to work in comfortable jobs. They should work more in home office jobs or flexible working hours. If women are in charge of the household, they can do this kind of work. Both men and women can choose jobs according to themselves” (I36, F, Vocational High School).

Regarding “women and men feeling the pressure of society in their occupational choices,” most students stated that the social segregation of men and women in professional life is dominant. Quotations for these opinions are presented below.

“I think this thinking in the professions is the usual traditional understanding. The fact that women were more in the background compared to the old life has been quite effective. Women did not participate in education and occupations where they remained in the background. When we look at the current situation, women are just starting their careers. When we look at it, we see that 60 to 70% of men still have careers. Some things seem closed to change, but there is an effort” (I29, M, Anatolian High School).

“I think it is about power. The doctor is a man; the nurse is a woman. Because the doctor is more important. A pilot is a man; a cabin attendant is a woman. For example, if we look at the general structure of the professions where women are in the majority, some things overlap with the stance of women in society. Looking at flight attendants, smiling faces, and communication are expected from women in social life. Alternatively, if you look at a soldier, strong, tough, these are behaviors expected from men” (I27, F, Vocational and Technical High School).

Based on these findings, students in vocational and technical high schools more clearly express that gender roles guide their career choices, whereas students in Anatolian and science high schools interpret these influences more in terms of individual interests and abilities. Female students in Imam Hatip and Anatolian high schools tend to choose occupations that align with societal expectations and caregiving responsibilities, while female students in science high schools consider professions that are both suitable for their interests and abilities and provide social status. Male students, on the other hand, prioritize providing for the family and high income across all school types; however, this tendency is more pronounced in vocational and technical high schools.

Table 4 presents the findings on the effect of gender roles on secondary school students’ reasons for choosing a profession.

**Table 4:** The effect of sexist job discrimination in society on students’ choice of profession.

Theme	Codes	Female (f)	Male (f)	Total (f)
Duties and responsibilities imposed on gender	Perception of responsibility in career choices	18	39	67
	Effect of domestic responsibilities	29	16	45
	Working conditions and gender discrimination in employment	22	17	39
Gender discrimination in professions	Gender-based motivations in choosing a profession	22	18	40
	The effect of gender perception on occupational choice and business life	27	32	59
	Gender-based inequality of opportunity	25	17	42

According to Table 4, students’ views on the effect of sexist job discrimination in society on occupational choice were categorized under two themes: “Duties and responsibilities attributed to gender” and “Gender discrimination in occupations.” Student views on “duties and responsibilities attributed to gender” were coded as “perception of responsibility in occupational choices” (*f* = 67), “effect of domestic responsibilities” (*f* = 45), “working conditions and gender discrimination in employment” (*f* = 39). “Perception of responsibility in occupational preferences” differs due to society’s expectations for men and women. It is thought that women’s emotional and empathic aspects are suitable for responsibilities in individual-oriented jobs, while men are successful in logic, technical, authority, and power-oriented jobs. In this context, perceptions of responsibility assumed according to gender may be determinative in occupational preference. In addition, gender-based domestic responsibilities are more effective in female students’ choice of profession (*f* = 29). In addition, the responsibilities attributed to gender in working conditions and employment in professions are considered more by female students (*f* = 22). Opinions were expressed that work life is perceived according to gender and that the responsibilities assumed accordingly affect employment opportunities. According to these views, the students expressed the problems that women and men may face in professions, and it was mentioned that women cannot work in every job due to the responsibilities brought by the domestic division of labor and their physical structure. It was stated that men are in more difficult working conditions due to their stronger structure and that they may face more oppressive attitudes at work. However, it was stated that women would not be exposed to the behaviors shown to men due to their sensitive nature.

“Women know that they will not go to places that require power. When we look at the point of finding a job, men usually work in factories, while women tend to work in desk jobs such as accounting and human resources. For men, bosses cannot yell at women, but they can yell at men and act more oppressively. While women are treated more carefully, men can be treated more easily” (I25, M, Anatolian High School).

The students’ views on the “effect of domestic responsibilities” on women’s choice of profession are concentrated on the fact that women’s socially accepted responsibilities in the family may also cause them to be unable to find a job. In addition, due to the domestic responsibilities imposed on women, they think they will not be able to show the necessary care and attention to their professions, negatively affecting their career planning. Some of these opinions are as follows.

“In business life for women, single and childless women who will not disrupt the work are mostly preferred” (I73, F, Science High School).

“Women cannot perform their professions at the desired level, they cannot concentrate on their work, they think about their family responsibilities, and therefore this situation reflects negatively on their promotions” (I28, F, Vocational High School).

According to the students’ views on “working conditions and gender discrimination in employment”, women are subjected to discrimination in processes such as recruitment and promotion. It was stated that most students internalized and adopted this situation. Female students, in particular, internalized this situation by accepting that they could not pay enough attention to their professional lives due to the injustice between men and women in the division of labor at home. In this context, they stated that women cannot be promoted at work because they do not pay enough attention to their work, due to the unfair division of labor at home.

According to Table 4, when students’ views on “gender discrimination in professions” are analyzed, they are coded as “gender-based motivations in choosing a profession” (*f* = 40), “the effect of gender perception on career choice and professional life” (*f* = 59) and “gender-based inequality of opportunity” (*f* = 42). The majority of the students think that professions have a gender. Students prioritize “gender-based motivations in choosing a profession” in this context. For students, professions such as nursing and teaching are considered female, while professions such as piloting, engineering, and police work are considered male. Some of the opinions about this situation are as follows.

“I do not think so, but our people do not see it that way. I think both men and women can do all professions regardless of gender. Kindergarten teachers and psychologists are more likely to be women. They prefer to go to a woman rather than a man to a psychologist” (I30, M, Vocational High School).

“If a woman chooses a profession deemed appropriate for women, her motivation will increase, she will develop more in a job where she is approved, her title will rise, and her path will be paved. Because everything that is not approved by society is short-lived, because what the majority wants usually happens anyway” (I2, F, Anatolian High School).

Among the opinions expressed here, the statements of interviewee 30 stand out. Although interviewee 30 started her sentence by saying that professions have no gender, she later stated that some professions are more suitable for women. Even though the interviewees thought they had a high level of gender awareness, it was determined from the answers given that this was not the case.

When the students’ views on “the effect of gender perception on occupational preference and professional life” were analyzed, it was determined that specific jobs were primarily associated with men or women. My opinions regarding this situation are as follows:

“I do not think professions should have a gender, but there is such a distinction in our society. However, with the mechanization of everything, I think both men and women can do any job. I think it is not right to genderize professions. When I say construction, I inevitably think of more men, or when I say cleaning, etc., I think of more women, which is not right, but yes, it exists” (I66, F, Science High School).

“I think professions have gender in society. This is a problem not only in our country but worldwide. When you look at it, jobs that require power and authority are seen as jobs, such as babysitting, etc. Such jobs are considered additional worldwide” (I15, M, Anatolian High School).

In the study, some students emphasized the “inequality of opportunity based on gender” in society and stated that they did not want to do professions appropriate for the type of secondary school they attended. Female students (*f* = 25) who chose secondary education institutions and departments according to the occupation they wanted to pursue, wanted to change their occupational preferences after school because they realized that jobs were divided into men’s and women’s jobs. This situation is predominantly found among female students studying at vocational high schools. Some of these opinions are as follows.

“Since the jobs are divided into male and female jobs, I thought that I would not be able to find a job in another field or my field, and I even encountered this problem many times while looking for an internship. For example, last year another friend of mine graduated from this school, studying informatics, and she was also my girlfriend. All the companies she applied to did not hire her because they wanted men” (I40, F, Vocational High School).

“One of our professors told us that they do not hire women civil engineers because they will be pregnant and will take maternity leave. After all, the woman always takes leave for childcare, and the father has no such responsibility. I have not seen any women who want to be a civil engineer” (I1, F, Anatolian High School).

When examining these findings, differences can be observed in students’ perceptions of gender roles and career choices according to school type. Vocational high school students more explicitly express that gender-based distinctions limit their career options, whereas science and Anatolian high school students recognize this perception but prioritize individual factors such as interests and abilities in their choices. In particular, female students in vocational high schools perceive their opportunities to work in certain professions as limited due to gender norms; female students place greater emphasis on family and domestic responsibilities, while male students focus more on income and prestige.

The research findings indicate that students’ career choices are influenced by existing societal stereotypes regarding female and male professions. Students tend to perceive certain occupations as more appropriate in line with societal expectations and gender roles; this, in turn, affects their own professional desires and choices. In particular, female students evaluate their options within the framework of the perception that certain professions are traditionally suitable for women or men. Thus, there is a direct relationship between students’ career choices and their beliefs about social stereotypes.

Furthermore, when the research findings are considered collectively, connections between the themes are evident. The preferences for “employment opportunities” and “desk-based work” shown in Table 2 are closely related to gender roles identified in Tables 3, 4. In this context, students’ views on career choices have been grouped into three distinct profiles based on common trends. These profiles are categorized as students who conform to social norms, students who are status- and achievement-oriented, and students who question gender equality.

Students who conform to social norms base their career choices on societal gender roles and expectations. Among female students, preferences for “desk-based work” and “workplaces with a majority of the same gender” are prominent, while male students prioritize “high income,” “prestige,” and the “breadwinner role.” Family guidance and the distinction between female and male professions are also defining features of this profile. Status- and achievement-oriented students include both female and male students who focus on individual success. Factors such as “employment opportunities,” “high income,” “promotion potential,” and “career development” are prioritized by these students. Although they are aware of societal norms, they believe that the primary determinant in career choice is individual skills and achievement. A small number of students who question gender equality take a critical stance toward linking professions with gender. This group, highlighted by the statement “professions should not have a gender,” argues that with technological advancements and changing social conditions, both genders are capable of performing any occupation. However, they also acknowledge that such gender-based distinctions still exist in society. These three profiles demonstrate that students’ views on career choices are not merely individual preferences but are shaped in diverse ways by societal norms, economic expectations, and perceptions of equality.

## 4 Conclusion and discussion

In the study, the effect of gender on occupational preferences according to secondary school students was evaluated according to the opinions of female and male students in the themes of factors affecting occupational choice, preferred working conditions, the effect of social stereotypes on occupational choice, duties and responsibilities attributed to gender, and gender discrimination in occupations.

According to the study, male students prioritize interest and talent in career choice, followed by prestige, employment prospects, career development, entrepreneurship, and income level. Their preference for careers with high development and earning potential reflects societal expectations of men and shows that they have internalized gendered social norms. However, this situation does not apply to all male students; it is also observed that some students prioritize different values in their career choices. Similarly, Buser et al. (2014) found that male students are more inclined toward prestigious, competitive, and STEM-related fields even with similar academic performance. In contrast, female students are primarily influenced by job availability, university entrance scores, personal interests, abilities, and family guidance, while earnings and career advancement are ranked lower. This suggests that family influence plays a more prominent role in the career decisions of female students. Supporting this, Abdullah (2025) and Eagly and Karau (2023) highlight men’s dominance in senior and managerial roles. Other studies (Abdullah, 2025; Köyüstü Erdeniz, 2017; Morgan et al., 2001; Olsson and Martiny, 2018; Özsoy, 2024; Ramaci et al., 2017; Terzioğlu and Aksöz, 2022; Truong and Duong, 2022) also emphasize that both genders behave in line with societal expectations in professional contexts.

The findings of the study indicate that a traditional patriarchal mindset significantly influences occupational preferences. It should also be noted that some students are aware of gender stereotypes but do not fully adopt or endorse them. Gender-based division of labor and the roles assigned to men and women are central in shaping career choices. While many participants stated that non-physically demanding professions are suitable for both genders, both male and female students agreed that women should prioritize their families, spouses, and children. Notably, the perception that motherhood impacts women’s career choices was shared by both groups, reflecting a traditional perspective among the students. However, it cannot be asserted that all students share the same view; some students have been observed to adopt more egalitarian perspectives. Although female students acknowledged that gender-based expectations in society contribute to inequality, they were also found to have internalized these expectations. These results are consistent with Olsson and Martiny (2018), who explored how gender stereotypes influence behavior. Similarly, Dahiya (2024) and Cuddy et al. (2015) highlighted the role of cultural values in internalizing gender norms and how women adopt these societal expectations. In this context, it is evident that gender roles shape career preferences, with both genders categorizing professions accordingly, and that the internalization of gender roles plays a critical role in occupational orientation and decision-making.

Societal expectations suggest that women pursue careers aligned with their perceived characteristics and maintain work conditions that do not interfere with domestic and social responsibilities. The belief among students that certain professions are more suitable for women and require competencies unique to them reflects the persistence of gender stereotypes. Nevertheless, a small number of students indicated that they did not agree with these stereotypes. Students generally think that career plans should align with gendered responsibilities—men as providers, women as caregivers, prioritizing family and children. These findings align with Koenig and Eagly’s (2014) study, which explains how gender roles influence perceptions of personal traits and occupational preferences. Similarly, Correll (2001) and Oswald (2008) demonstrate that cultural beliefs about gender shape perceived competencies, often outweighing actual ability. Over time, the responsibilities expected from men and women have been linked to assumed innate traits: men are seen as assertive, risk-taking, and financially capable, while women are viewed as emotional, nurturing, and suited to caregiving roles.

The research indicates that women are perceived as more disadvantaged in working life due to gender roles, particularly their domestic and motherhood responsibilities, and passive positioning in social life. However, this finding is derived from the students’ perspectives and is limited in terms of generalizability. Both male and female students agree that working women struggle to balance home and work duties, limiting their ability to focus on their careers fully. Social expectations and responsibilities hinder women’s success and active participation in professional life. A dominant view is that gender stereotypes cause significant challenges for women, including limited promotion opportunities and employer policies that, while aiming to accommodate domestic roles, may contribute to unemployment or exclusion from managerial positions. These findings are supported by Aydoğan (2024) and Smith (2012), who emphasize that traditional gender norms and prejudices reinforce systemic discrimination, hindering women’s career advancement. Students’ belief that women should structure their careers around domestic duties reflects an awareness of these systemic challenges. Gendered expectations continue to influence all stages of social and professional life.

In the current study, students may encounter different attitudes and behaviors, such as the opposite sex in a profession, attributed to women or men within the framework of gender stereotypes. In the same way, they stated that they would prefer a female employee in a profession considered suitable for women, and that they could trust a male employee in a profession considered suitable for men. Students believe they will be more successful within the social boundaries drawn in working life. In similar studies, it has been determined that individuals tend to associate professions with gender and that these tendencies emerge at conscious and unconscious (implicit) levels (Kılıç et al., 2014; Ramaci et al., 2017; Simpson, 2004; White and White, 2006). In this context, it can be said that students act from a traditional perspective and do not have an egalitarian attitude. However, it is also observed that some students oppose gender-based distinctions and exhibit a more flexible approach.

Although female students expressed discomfort with gender-based occupational divisions, the study revealed that environmental factors and gender norms also influenced their views. Many reported concerns about being perceived as less successful or unsuitable professionally. Spieler et al. (2020) similarly found that environmental influences and gender stereotypes shape women’s career preferences and anxieties. Professions perceived as “female jobs” were seen as easy, low-status, and often extensions of motherhood. Care, service, and emotional labor roles in the public sphere were attributed to women. At the same time, job conditions and work environments contributed to classifying professions as either male or female. Students believed that women should pursue jobs that would not interfere with domestic duties, whereas men should choose careers suitable for providing for the household. This reflects internalized gendered responsibilities. Research by Jaoul Grammare (2024), Tabachnikova and Vinokurova (2024), and Csernus and Siegler (2024) emphasizes that students tend to choose careers aligned with traditional gender expectations, which limits occupational choices. Although female students supported the idea of gender equality in professions, their career aspirations remained within traditional boundaries. The persistence of gender-based division of labor demonstrates that traditional ideologies still influence students’ thinking, despite modern representations of gender.

These findings indicate that students’ perceptions of gender roles largely reproduce existing patterns. However, this reproduction is the result of a structural process based on internalizing societal expectations of femininity and masculinity from an early age, rather than individual choices. Students’ statements regarding career choices reveal that gender shapes not only individual attitudes but also concepts such as “appropriate occupation,” “success,” and “responsibility.” This suggests that young individuals tend to conform to societal expectations rather than critically question them. In other words, although students do not directly challenge gender-based patterns, these patterns limit their professional aspirations and identity perceptions. Accordingly, the research findings show that gender inequality is reproduced not only in adulthood but also at the stage of career choice, and this process is indirectly reinforced by educational institutions, families, and cultural values.

The findings of the study indicate that there are certain relationships among the emerging themes. In particular, there is a connection between the influence of gender stereotypes on career choices and expectations regarding working conditions. For instance, the tendency of female students to prefer occupations compatible with home and family life demonstrates that social roles shape not only career selection but also preferences related to professional life. Additionally, different attitudinal profiles among students were identified. A significant portion of students (approximately 45%) adhere to social norms and gender roles in their career choices, whereas another group (around 40%) focuses more on individual achievement and economic gain. A smaller group of students (about 15%) critically questions the gender-based segregation of occupations. Although the number of students who explicitly question gender-based occupational distinctions is small, they provide an important counter-narrative. This profile, which can be referred to as “gender-questioning,” is noteworthy for its views that challenge traditional social norms and argue that occupational identity should not be predetermined by gender. However, in the statements of these students, a dual structure is observed: while they conceptually reject social patterns, they simultaneously acknowledge the existence of these patterns in practice. This indicates that adolescents both reproduce and, to some extent, question societal expectations. Therefore, the tendency to question gender equality emerges not yet as a dominant mindset but as an awareness shaped by social change, education, and digital interactions. Furthermore, the overlaps observed among themes such as “domestic responsibilities,” “gender-based discrimination,” and “working conditions” demonstrate that students’ perceptions of gender roles are multilayered. In particular, female students’ emphasis on domestic responsibilities is directly related to perceptions of limited career advancement opportunities. Similarly, male students’ emphasis on the “breadwinner” role creates a hierarchical structure in occupational choices at both economic and symbolic levels, reproducing gender-based labor divisions. In this context, there are permeable boundaries among the themes of “responsibility,” “discrimination,” and “professional opportunities.” Thus, career choice reflects not only individual interests and abilities but also the entrenched societal norms that determine who is directed to which occupations under which conditions. An in-depth examination of these diverse profiles can contribute to a more comprehensive understanding of the impact of gender roles on individuals’ professional orientations.

Overall, social gender roles significantly influence the career choices of secondary school students. Male students tend toward prestigious and high-income professions, while females are inclined to choose jobs aligned with societal norms and family responsibilities. This reinforces occupational gender inequality. Students acknowledge that gender stereotypes in occupational choices hinder social equality.

## 5 Limitations and suggestions

This study was limited to secondary-school students in Sakarya, Turkey. Future research could expand to multiple regions and compare Turkish students’ views with those of students in different cultural or national contexts. The study focused on traditional gender roles and career choices via student statements; however, the effects of changing gender norms, digitalisation, feminist movements, and post-COVID-19 labor-market transformations were not sufficiently addressed not fully explored, although they may significantly shape perceptions of gender roles (Küçük and Çoksan, 2023). Broader investigations should consider additional variables and socio-cultural factors affecting career choice.

Underpinning theoretical frameworks such as social learning theory and gender role socialization Theoretical perspectives such as social learning theory and gender role socialization posit that children and adolescents internalize gender norms via family, school, peers, media, and institutional contexts (Sakallı Uğurlu et al., 2018). Empirical evidence from Turkey shows that childhood experiences in family and school life are significantly associated with occupational choice capability and that family sexism undermines career-choice freedom (Başfırıncı et al., 2019). These findings support the view that gender stereotypes are reproduced early and influence occupational aspirations.

In terms of policy and structural change, specific recommendations for Turkey include:

Expanding access to public/community-based childcare services can relieve the disproportionate care burden often placed on women and enable more egalitarian career choices. For example, in 2021, Turkish municipalities and international organizations signed a memorandum to enhance childcare services for working women (Turkish municipalities and EBRD/ILO, 2021).

Strengthening and broadening paternity leave and parental-leave rights is essential. A recent study shows how childcare and parental-leave regimes reinforce or challenge gender equality (Özkan, 2024).

Enforcing and promoting anti-discrimination legislation and practices in education and employment is key to dismantling structural barriers. Empirical research indicates that women in STEM or caregiving professions face gendered institutional barriers and discrimination (Durmaz Aslan, 2020).

Media campaigns, educational curricula, and public-awareness programs should promote gender-neutral occupational representations and challenge traditional stereotypes of “women’s jobs” vs. “men’s jobs” (Sakallı Uğurlu et al., 2018).

Family- and school-based interventions are essential: supporting parents and teachers to guide students’ career choices based on interest and ability rather than gender expectations (Küçük and Çoksan, 2023).

In sum, policymakers, educational institutions, and civil society in Turkey should adopt a multi-level strategy: structural reforms (childcare, leave policies, labor regulations), institutional reforms (school curricula, teacher training, career guidance), and cultural interventions (media, family education, role-model visibility). Without such integrated and evidence-based action, the internalization and reproduction of gender stereotypes will continue to limit students’ occupational aspirations and perpetuate gender-based divisions in the labor market.

## Data Availability

The original contributions presented in the study are included in the article/supplementary material, further inquiries can be directed to the corresponding author.
